# Physico-Mechanical Properties of the Poly(oxymethylene) Composites Reinforced with Glass Fibers under Dynamical Loading

**DOI:** 10.3390/polym11122064

**Published:** 2019-12-11

**Authors:** Stanisław Kuciel, Patrycja Bazan, Aneta Liber-Kneć, Aneta Gądek-Moszczak

**Affiliations:** 1 Faculty of Materials Engineering and Physics, Institute of Materials Engineering, Tadeusz Kosciuszko Cracow University of Technology, Al. Jana Pawła II 37, 31-864 Cracow, Poland; patrycja.bazan@gmail.com; 2 Faculty of Mechanical Engineering, Institute of Applied Mechanics, Tadeusz Kosciuszko Cracow University of Technology, Al. Jana Pawła II 37, 31-864 Cracow, Poland; aliber@pk.edu.pl; 3 Faculty of Mechanical Engineering, Institute of Applied Informatics, Tadeusz Kosciuszko Cracow University of Technology, 31-864 Cracow, Poland; aneta.moszczak@gmail.com

**Keywords:** composites, poly(oxymethylene), fibers, properties, fatigue

## Abstract

The paper evaluated the possibility of potential reinforcing of poly(oxymethylene) (POM) by glass fiber and the influence of fiberglass addition on mechanical properties under dynamic load. Four types of composites with glass fiber and another four with carbon fiber were produced. The fiber content ranged from 5% to 40% by weight. In the experimental part, the basic mechanical and fatigue properties of POM-based composites were determined. The impact of water absorption was also investigated. The influence of fiber geometry on the mechanical behavior of fiber-reinforced composites of various diameters was determined. To refer to the effects of reinforcement and determine the features of the structure scanning electron microscopy images were taken. The results showed that the addition of up to 10 wt %. fiberglass increases the tensile properties and impact strength more than twice, the ability to absorb energy also increases in relation to neat poly(oxymethylene). Fiber geometry also has a significant impact on the mechanical properties. The study of the mechanical properties at dynamic loads over time suggests that composites filled with a smaller fiber diameter have better fatigue properties.

## 1. Introduction

Polymer composites are classified as a group of construction materials. It has been found that the addition of fillers in the form of fibers and particles to the polymer matrix advances the mechanical characteristics of polymeric materials. The strength and stiffness of the polymeric materials can be effectively developed by means of reinforcing fibers. Glass fiber and carbon fiber are widely used as reinforcement in a thermoplastic matrix because they provide a great balance between mechanical properties and economic features [1].

Carbon fibers are produced mainly by polyacrylonitrile as a result of pyrolysis and their properties are primarily affected by the production parameters [2,3]. The main advantages of appliance carbon fibers to reinforce polymeric matrices are low density, good mechanical properties, thermal and electrical conductivity, in addition, these fibers can suppress vibrations and have low absorption of X-rays. Carbon fibers as a reinforcing element of composite materials based on polymer matrices have been increasingly applied in recent years. Carbon fiber-reinforced polymers replace traditional materials and nowadays are used to make yachts and boats masts, bicycle frames or arrowheads. Additionally, these fibers are employed for the production of heat-resistant and non-flammable fabrics, as well as heating fabrics powered by electricity [2,3,4,5]. Many scientific papers present the advantages of introducing carbon fibers to thermoplastic materials. Carbon fibers are not only lightweight or provide very good strength properties, but also have a positive effect on tribological properties, and therefore they also can be successfully used as a reinforcement in ceramic matrices [6,7,8,9,10,11,12,13,14,15,16,17].

Carbon fibers are not only one fiber introduced in composites which provide good mechanical properties. Currently, about 60% of produced glass fibers are applied to make composites on polymeric matrices, even though they have insignificantly lower physical and mechanical properties than carbon fibers. Glass fibers are characterized by low elongation and high modulus of elasticity. Attention should be paid to good dielectric properties, namely the fibers have low values of relative permittivity and dielectric loss factor. A valuable advantage of glass fibers is the very good wettability of the polymers, and hence the possibility of forming a strong bond at the polymer/glass interface. The mechanical properties of fiber-reinforced composites improve as the length of reinforcing fibers increase, while the strength of a single glass fiber depends on its diameter. If the fiber diameter is larger, there is a higher probability of material defects and damages describe in literature as micronotches and microcracks [18].

Many groups of researchers conduct research on polymer composites with glass fiber. It has been revealed that glass fibers successfully strengthen polymers causing better strength properties, higher resistance to creep, and fatigue, as well as provide greater dimensional stability [19,20,21,22].

POM is a thermoplastic material with a high degree of crystallinity. This polymer is obtained from formaldehyde by homopolymerisation (POM-H) or copolymerization with cyclic ethers (POM-C). The mechanical properties of acetal materials depend on the content of the crystalline phase, the value of which reaches 70%–75%. The high degree of crystallinity determines the high rigidity of the polymer, which implies high resistance to fatigue and creep stresses. This feature allows exert of poly(oxymethylene) under variable load conditions. The high proportion of the crystalline phase is also the reason for high hardness, better mechanical strength, abrasion resistance, and significant resistance to elevated temperatures. POM is one of the strongest and stiffest thermoplastics with very good dimensional stability [23].

Polyoxymethylene in scientific works has been modified with many fillers. For example, addition micro and nano polytetrafluoroethylene (PTFE) particles reduce the friction coefficient and abrasive wear of the composite. Studies show that addition of PTFE to the material enhances tribological properties through formation of a PTFE-based transfer film yet resulting in decrease mechanical properties [24,25,26,27,28,29]. The usage of glass fiber in POM composites leads to a significant improvement in strength and stiffness of the element and decreases wear, as well as sound attenuation, but enlarges surface roughness and raises coefficient of friction [28,29,30,31]. The fibrous filler, which increases the strength and wear resistance, reduces the coefficient of friction in the polyoxymethylene composites is aramid fiber [6,24,32]. Carbon fiber incorporated into the POM matrix not only provides better strength properties but also positively effects on the abrasion resistance. With increasing fiber content, the coefficient of friction and wear are reduced [6,32,33,34]. Subsequent fillers use to improve the properties of polyoxymethylene composites are low density polyethylene (LDPE) and wood grain, copper particles or basalt fibers, all of these additives modify POM composites in the direction of develop its strength, thermal, and tribological properties [20,26,27,35,36].

In this work, polyoxymethylene composites reinforced with glass fiber (cut roving, with diameter of 10 and 13 µm, and length about 150–200 µm) and carbon fibers (diameter of 7 µm and length about 3 mm) were analyzed. Research showed the effect of used reinforcements on strength properties and behavior of composites under dynamic load. This is a very relevant and up-to-date topic because the use of composites is increasing in structural applications, and many composites have been developed relatively recently and there are uncertainties about the long-term performance of these composites and how they will work in cyclic fatigue loads. Fatigue is a progressive and local structural damage that occurs when the material is subjected to a cyclic load. Knowledge of fatigue resistance of materials is very important because it allows to definite a safety working conditions of materials.

## 2. Materials and Methods

### 2.1. Materials

The subject of the research was polyoxymethylene composites with the trade name Tarnoform 300 (a standard variety that quickly solidifies and it is intended for injection), Zakłady Azotowe S.A., Tarnów, Poland with different fiberglass contents (cut glass roving ER5001F Krosglass S.A., Krosno, Poland with diameter of ø = 10 µm or ø = 13 µm and length l = about 150–200 µm) in an amount of 10%, 20%, 30%, and 40% of fibers by weight. The selected properties of glass fiber composites were contrasted with Tarnoform 500 composites (a variety with a higher melt index), Zakłady Azotowe S.A., Tarnów, Poland with carbon fiber addition (Zoltek PX35, Toray Group USA, Bridgeton, MO, USA with diameter about ø = 7 µm and length l = about 3 mm) in the amount of 5%, 10%, 20%, and 30% of fibers by weight. The samples were made by injection method after filling the granules with fibers on the compound line at Zakłady Azotowe S.A. (Tarnów, Poland).

The basic parameters of the injection molding process of dumbbell samples, bars, and other details were:

- mold temperature was set between 60 and 120 °C (depending on the desired dimensional tolerance),
- pressure in the clamping phase was in the range of 60 to 180 MPa,
- closing time was selected experimentally as a function of the workpiece mass,
- alloy temperature was between 180–230 °C.

Drying of Tarnoform before processing is not necessary if the transport and storage process were carried out as it is required, i.e., transport by covered transport means, storage at a temperature not higher than 50 °C in dry rooms, far from heat sources. Long-term storage or moisturizing cause an increase in the water content in the granulate to approx. 0.2%, which may cause difficulties in processing, resulting in yellowing of the material during processing and poor appearance of the surface of the fittings (silvery streaks, micro blisters). In this case, it is necessary to dry Tarnoform^®^ at a temperature of 100–120 °C for 2–3 h (for shelf dryers, the thickness of the plastic layer should not exceed 3 cm). If the material is stored at a temperature lower than 20 °C, it should be brought it to ambient temperature before introducing the material.

### 2.2. Method of Testing

Basic physical and mechanical tests of poly(oxymethylene) and its composites were carried out. Density was determined by the hydrostatic method (scale RADWAG WAS 22W, Radom, Poland). Sorption of water (20 °C) was determined after 1, 7, 30, 240 days of soaking, according to PN-EN ISO 62:2000. Vicat softening temperature (VST) was tested according to ISO 306 under 50 N loading and with 50 °C/h heating rate using CEAST machine (Instron, Norwood, MA, USA). Specimens for mechanical testing were conditioned at 23 °C/50% relative humidity for at least 80 h according to ISO 291 for test room conditions. The mechanical properties were identified by a static tensile test (PN-EN ISO 527-1:20100) and the three-point flexural test (PN-EN ISO 178:2011) using universal testing machine Instron type 4465 (Norwood, MA, USA) with a measuring range up to 20 kN. Examination under wide range of temperature was performed using Instron temperature chamber (−80–300 °C). The test speed was set to 10 mm/min. Charpy impact test (PN-EN ISO 179-1:2010) was examined on unnotched and notched specimens using a Zwick HIT 5.5P (Zwick Roell Group, Ulm, Germany). The stress-controlled fatigue tests for tensile were conducted at 23 °C on an Instron 8511.20 hydraulic testing machine Instron, (Canton, MA, USA). The tests were performed at constant minimum and maximum stress for a specified number of cycles (5000 cycles), and then the maximum stress was elevated to continue the fatigue test for the next 5000 cycles. This was repeated until breaking. Thus, during the tests, the amplitude was changed every 5000 cycles by increasing the maximum stress level by 5% tensile strength of the material, starting from 30% of tensile strength for each material. The preload was set at a minimum level of 2 MPa for easier calculations so that the hysteresis loops do not pass through the zero level. All fatigue tests were carried out at a 5 Hz cyclic frequency with a sinusoidal waveform. The surface temperature of the samples was measured using a pyrometer. Hysteresis loops were recorded, and then the maximum strain and dispersed energy were calculated for each level of maximum stress. To determine fatigue strength based on strain-stress curves or dissipated energy-stress curves, lines tangent to curves were drawn. The average stress values at the tangential intersection for these two curves was the fatigue strength value of the modified Lehr method used.

Thermal analysis by differential scanning calorimetry (DSC) was led using a Q2000TA Instrument apparatus, (New Castle, DE, USA). The specimens for the DSC test were prepared in the same way as the samples for mechanical characterization. All samples about 8–12 mg were heated from −80 °C to 300 °C with the heating rate of 10 °C·min^−1^ under helium atmosphere and kept at this temperature for 5 min to remove the thermal history. The degree of crystallinity was calculated on the basis of the sample’s heat-melting (endothermic peak) and the heat of melting of the fully crystalline sample. The calculations were made using the following formula:

(1) $$w_{cx}=\frac{ƩI_{c}}{ƩI_{c}+ƩI_{a}}$$

where:

*W_cx_*—degree of crystallinity
Ʃ*I_c_*—area of peaks derived from the crystalline phase
Ʃ*I_a_*—area of peaks derived from the amorphous phase

Diffraction studies were realized on a Bruker D8 X-ray diffractometer (Billerica, MA, USA) with Cu Kα radiation (λ = 0.14178 nm) using a parallel beam obtained by reflection of X-rays coming out of the lamp in a parabolic multi-layer mirror (Gbel Mirror). Diffraction images were recorded in Ɵ–2Ɵ geometry. Soller slits were used to improve the resolution. The monochromator was not applied on the reflected beam. The tests were carried out at room temperature. Samples made by injection were in the form of cuboids with sides 10 × 10 mm and height 2 mm.

The microstructure observations were made on the gold-sputtered tensile-test fracture surfaces. A thin gold layer was covered in order to avoid electrostatic charging during SEM analyses. The micrographic images were taken in high vacuum mode with 10 kV accelerating voltage and 13.7 mm working distance using a Scanning Electron Microscope JEOL JSN5510LV (Peabody, MA, USA) with computer software for image analysis (Aphelion). The values were obtained from an average at least of 5 specimens.

## 3. Results and Discussion

### 3.1. Physical and Mechanical Properties of Poly(oxymethylene) Composites with Glass and Carbon Fibers

Table 1 presents a comparison of the processing properties and density of tested composites. While the fiber content rises, the density of composites also grows. The melt flow rate decreases with fiber content increases. Glass fiber composites are characterized by a higher melt flow rate with increasing fiber content compared to carbon fiber composites.

**Figure 1:**
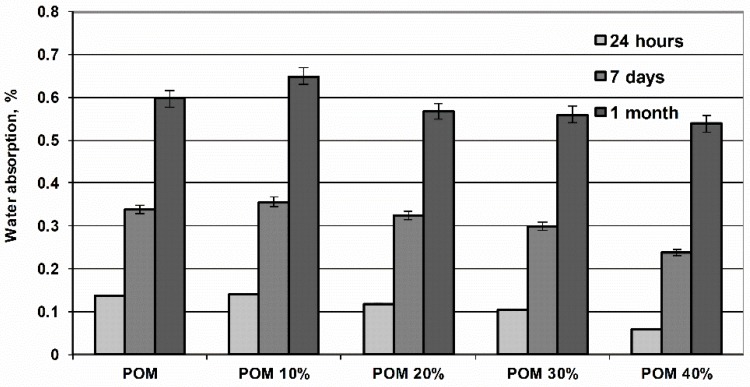
Water absorption of poly(oxymethylene) compositions in relation to fibers content and incubating time in water.

The water absorption of poly(oxymethylene) composites (Figure 1) elevates over time until the maximum level for POM (about 0.6%) is obtained, which is comparable to the maximum values reported in the literature. The addition of fibers reduces the ability to absorb water especially in the initial periods (up to 7 days), which indicates the possibility of water penetration through the fiber-polymer interface, especially at longer exposure time.

**Figure 2:**
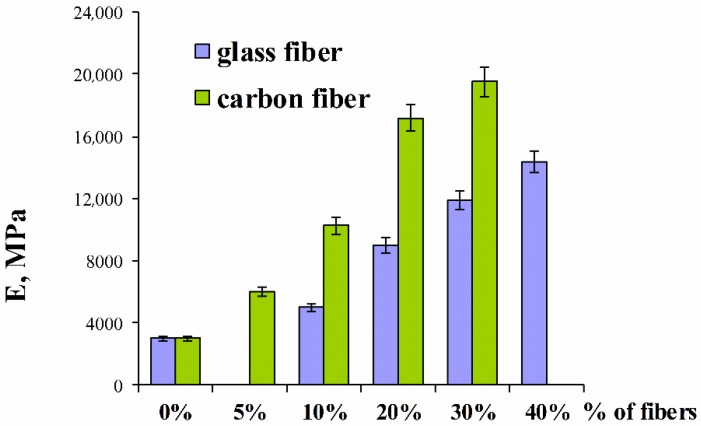
Relation of changes in tensile modulus values for different mass proportions of glass and carbon fiber in a composite.

Figure 2 compares the values of tensile modulus for poly(oxymethylene) composites with different content of glass and carbon fibers. Noteworthy gain in the modulus of elasticity of carbon fibers composites can be observed in relation to composites filled by glass fibers. In the case of POM with glass fiber, the elastic modulus is almost doubled between levels of 10 and 20% by weight of fiber. A further increase of filling does not cause such a strong growth any more. This may suggest that the addition of approx. 25% glass fiber is enough to enhance the stiffness of most products.

**Figure 3:**
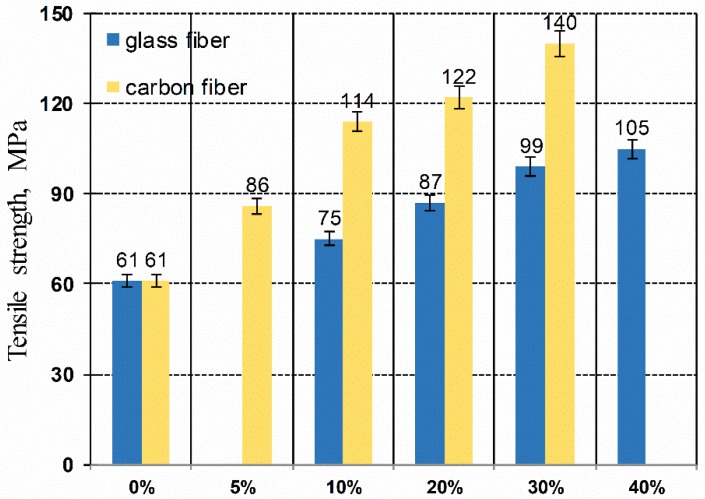
Comparison of the average values of tensile strength of POM composites filled with glass and carbon fiber with different mass fraction.

The connection of tensile strength of different composites is presented in Figure 3. Results demonstrate constant increment in tensile strength while content of glass fiber increases, yet it is not as significant as for carbon fibers, which may announce lower adhesion of glass fiber to the poly(oxymethylene) matrix than bonding between carbon fiber and POM, which may be related to the diameter and the length of the fibers. Adhesion is caused by forces occurring at the fiber/matrix interface. Adhesion strengths are physical forces resulting from the chemical structure of the polymer matrix and the filler forming the composite. They can also be chemical actions that work on the principle of functional group similarity. The properties of the composite depend on the behavior of macromolecules in thin layers on the surface of the filler. The flexibility of the macromolecule chain and changes in the conformation of the macromolecules affect the amount of adhesion, since they largely determine the number of contact points between the macromolecule chains and the fillers. Using longer carbon fiber with a smaller diameter (7 µm), the quantity fraction of these fibers is greater than while using glass fibers with a larger diameter, which results in a larger contact surface between the fiber and the matrix.

The modulus of elasticity determined in the bending test (Table 2) changes evenly with the increase of the fiber fraction and shows slightly lower values than the tensile modulus, which is a characteristic feature of most polymer materials, the reason of this behavior is their better orientation and strengthening during stretching (This phenomenon is related to the injection process. During the flow of the material in the injection mold, the fibers are oriented in the direction of flow of the molten mass. Testing standard test samples in a static tensile test, the fibers are arranged along the sample axis. By stretching the sample, the forces occurring during the test are consistent with the direction of the fibers and the load can be transferred from matrix to reinforcement. In the case of bending tests, shear forces and forces perpendicular to the fiber axis separate the matrix from the fiber surface. The ends of the fibers introduce additional stresses and in these places the material is the weakest. Hence the differences in the tensile and bending modules [37]).

Comparing changes in energy is needed to obtain maximum force, energy decreases with the fraction of fiber increases, and also diminishes for the modification of poly(oxymethylene) with carbon fibers. The deformation at the break declines with the proportion of fillers raises from the initial value of 10% of the filler in the composite. This suggests the need for some plasticization of the poly(oxymethylene) matrix. The softening property of plasticizers consists in reducing the intermolecular interactions of polar forces (van der Waals) attracts macromolecules to each other, reducing intermolecular friction with each other, increasing the free volume in the polymer matrix, reducing the brittleness temperature, all of which increase the mobility of macromolecules, increases polymer flexibility, facilitate processing and increase the ability to introduce fillers.

Notched impact strength determined in the Charpy test remains almost constant, only marginally increases with the amount of filler increases. This may be due to the fact that its value is mainly affected by the impact strength of the polymer matrix, which is not much different from the impact strength of the fibers.

**Figure 4:**
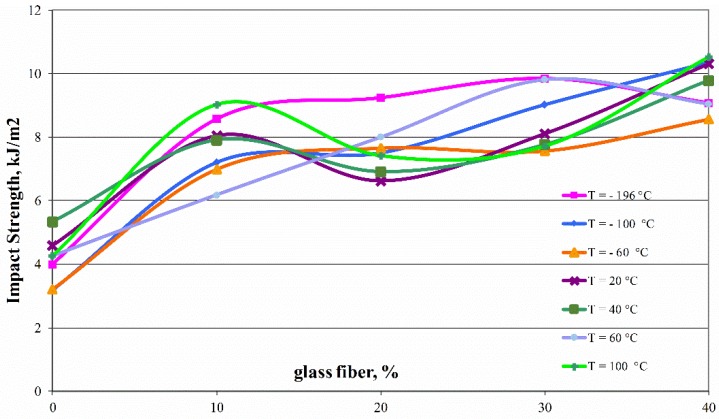
Comparison of notched Charpy Impact Strength (type V) for poly(oxymethylene) composites with different glass fiber fraction depending on the temperature of the determination.

The notch impact measurement results conducted at a wide temperature range from −196 to +100 °C are shown in Figure 4. They show that even a 10% fiber addition results in an almost twofold expansion in impact strength, which is still lightly increasing for a higher proportion of fiberglass in the composite. This dependence is linear for extreme temperatures, showing a slight decrease in intermediate temperature range for composite with 20 wt %. of fibers. Changes in the impact value indicate a change in the fracture character from plastic to brittle, which is associated with tearing the macromolecule in the crack plane. These changes are influenced by factors such as a decrease in adhesion between the reinforcement and the matrix, irregularity in the structure of the composite, cracks between crystallites, formations of micronotches during processing and the associated shrinkage of the material, the presence of inclusions as well as the size of the reinforcing phase or depolymerization of polyoxymethylene at elevated temperatures. The results of impact tests indicate high impact resistance of components made of poly(oxymethylene) composites. Despite of the increase in fibers content, no negative effects of micronotches or structure changes were observed, which suggests good cooperation between components in a wide temperature range.

### 3.2. Impact of Variable Loads over Time on Dynamic Properties and Changes in Structure

The aim of this research was to evaluate the impact of time-varying loads on selected mechanical properties of poly(oxymethylene) composites with the addition of glass fiber. The dynamic tensile tests were carried out at one 5 Hz frequency level for 60,000 cycles. A dynamic force was applied from 0.1 to 0.6 of the average value of the maximum force obtained in static tensile tests for POM composites with 10, 20, 30, and 40% fiberglass. The values of these forces and the values of the adopted average modulus of elasticity (for the calculation of initial strains) are shown in Table 3. The obtained results show that neat POM has a much smaller ability to dissipate mechanical energy and filling it with even a small (10%) amount of fiber causes a significant increase in dissipated energy. Interestingly, a higher amount of fiber (above 30%) improves even more the possibility of dissipating this energy. This proves that despite the slightly worse adhesion of glass fiber to the polymer matrix it is advisable to create composites based on poly(oxymethylene), not only for improving strength and stiffness, but also increasing their ability to dissipate energy. It should be also considered the additional possibility of increasing the adhesion of glass fibers to the polyoxymethylene matrix, e.g., by adding silane media, which contains active functional groups enabling chemical connection between the components. Silane compounding agents are widely used not only in the case of thermoplastic polymer composites but also in the case of thermosets reinforced with fibers [38,39,40]. Another option is to prepare the surface of the fiber to strengthen the mechanical adhesion, or if the injection process molding is being considered, the alternative is to control the process of shrinkage (increasing the content of the crystalline phase) to provide a better mechanical connection between the components.

**Figure 5:**
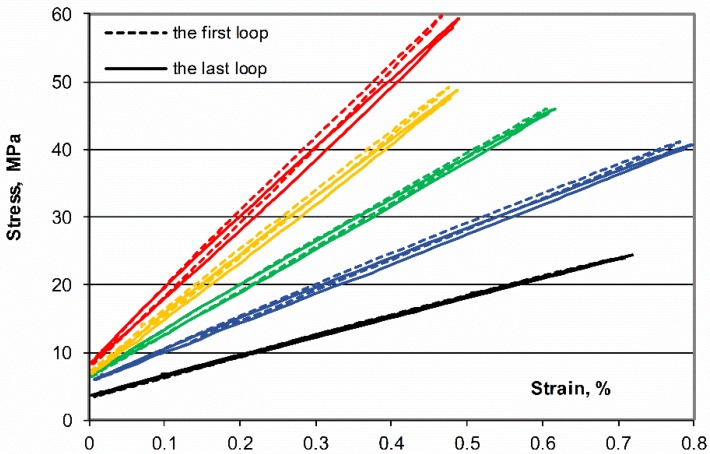
Examples of changes in the position of the first and last mechanical hysteresis loop (60,000 cycles, 5 Hz) for loads in the range 0.1–0.6 P_max_ of POM composites with different fiber content.

**Figure 6:**
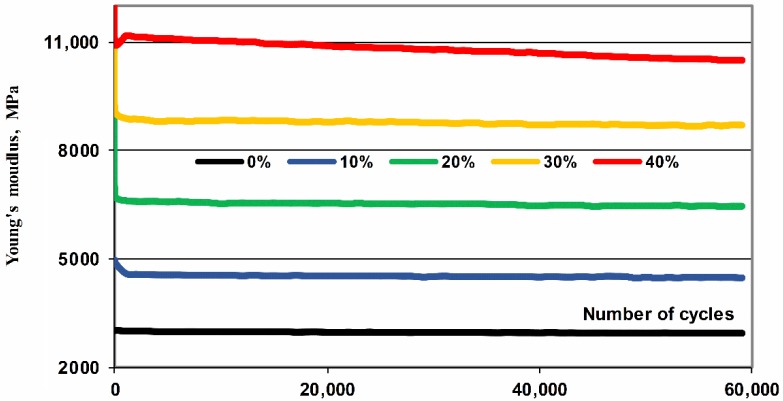
Comparison of the secant modulus of elasticity calculated in each loop (60,000 cycles, 5 Hz) of POM composites depending on the subsequent cycles for different mass fraction of glass fiber (10%–40%).

The comparison of the first and last recorded mechanical hysteresis loops is presented in Figure 5. With rise in fiber content (10–40%) the ability of composites to carry increasingly high loads and dissipate mechanical energy increase but displacements decrease. The comparison of the secant modulus of elasticity, presented in Figure 6, indicates a certain drop in their value in the initial phase of fatigue loads (up to 1000 cycles) and then their stabilization. The one exception is the most stressed composite with a 40% fiber content, whose modulus hardly noticeable decreases for subsequent loading cycles, testifies to the progressive process of its fatigue process.

**Figure 7:**
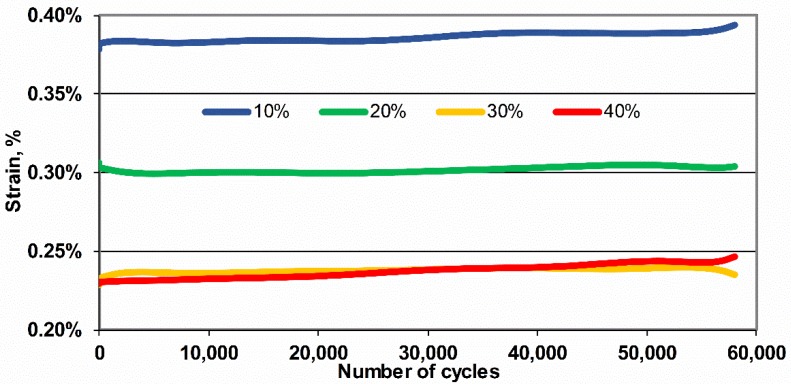
Changes in average strains calculated in each loop (60,000 cycles, 5 Hz) for tested POM composites with the increasing fraction of glass fiber.

Figure 7 presents the changes in average deformations calculated in each loop for the tested composites with the heighten fraction of glass fibers. Slight effects of dynamic creep with the increasing number of cycles and no significant difference in strain between the composites with 30 and 40% of the fiber content in the composite can be seen, which may indicate a dominant contribution of fatigue brittle failure in high fiber content composites.

**Figure 8:**
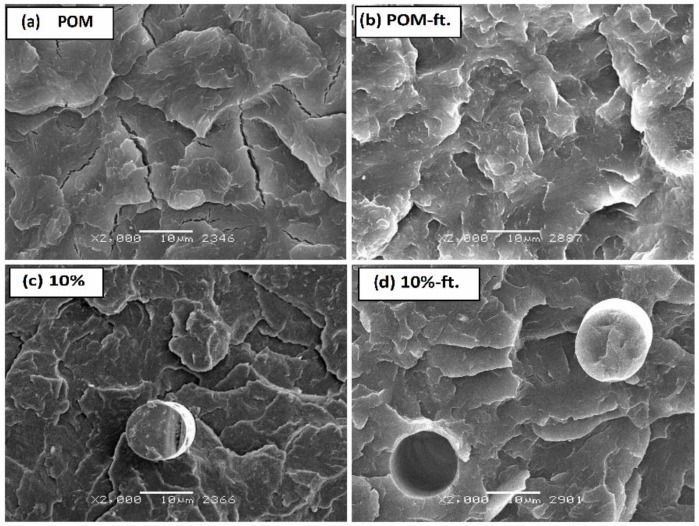
SEM microscopic images of POM and its composite with 10% fiberglass: (**a**) neat POM before fatigue test, (**b**) POM composite with 10 wt %. glass fibers after 60,000 cycles of time-varying loads, (**c**) POM composite with 10 wt %. of fiberglass before fatigue test, (**d**) POM composite after 60,000 cycles of time-varying loads.

In Figure 8, SEM pictures show changes in fracture surface of samples (broken in N_2_) caused by fatigue loads. A grow in the share of a ductile fracture, cracking, and strong fragmentation of spherulites can be noticed. Figure 8a,b show the changes and first surface deformations of the crystalline groups in POM caused by the fatigue of the polymer matrix, while Figure 8c,d show surfaces of deformation of the crystalline groups in the POM composite with 10% glass fiber (ø 13 μm), with the characteristic “voids” after pull out fiber process.

**Figure 9:**
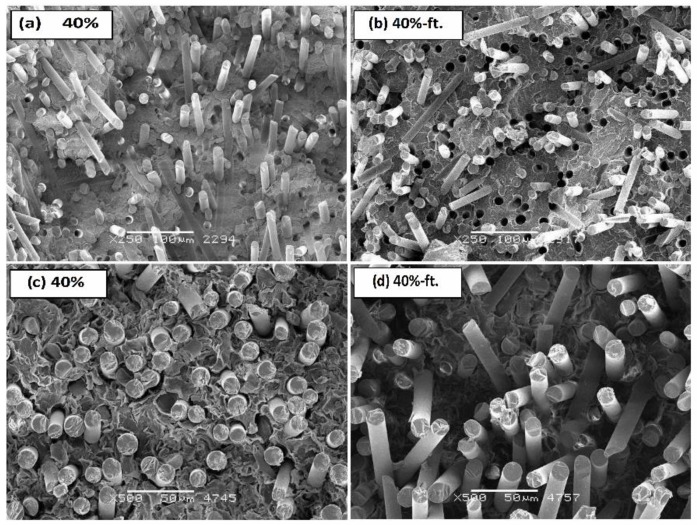
Microscopic images of SEM: (**a**,**b**) breakthroughs obtained at ambient temperature, and (**c**,**d**) etched samples for POM composite with 40%wt. of glass fiber content before and after (ft.) 60,000 cycles of variable load over time (5 Hz, range 0.1–0.6 P_max_).

Figure 9a,b show photos of non-etched poly(oxymethylene) composite samples with 40 wt %. fiberglass at 250× magnification. Figure 9c,d show the microstructure after etching at higher magnification (500×). Lower image magnification shows the distribution of the reinforcement in the polymer matrix, and the higher magnification shows the interfacial region between two phases with more details. In the image of the microstructure in Figure 9a, fibers with a diameter of about 13 μm can be observed, which are evenly distributed with random orientation in the polymer matrix. There are some holes observed on the fracture surfaces because of the fiber pullout, that indicates both the fiber pullout and fiber breakage are predominant mechanisms of fracture. Moreover, the surfaces of fibers look smooth, and the polymer matrix does not wrap around the fiber which point to a moderate interfacial adhesion between the matrix and fibers. Figure 9b shows a picture of the composite microstructure after fatigue tests. A large (about five-fold increase) effect of pulling the fiber out of the polymer matrix as a result of progressive fatigue processes is visible. The images of the etched microsection of composites (Figure 9c,d) show the plate-like structure of spherulites and faster leaching of the amorphous phase, the amount of which increases under the influence of fatigue loads.

### 3.3. Influence of the Fiber Geometry on the Properties of Poly(oxymethylene) Composites

The geometry of the reinforcing elements is extremely diverse, the fibers can be short, long, and continuous. The right choice stands on the specific application and the specific required properties of the product (e.g., anisotropy of properties, required relationships between stress and strain) often dependents on working conditions. Strengthening with fibers is usually more effective than particle reinforcement, and those, used to strengthen composites based on thermoplastics do not exceed 15 μm in diameter. The larger and more developed fiber surface and the better adhesion to the polymer matrix (the important role is played by its polarity), the better the strength properties of the entire composite. The fiber length of glass fiber is in the range of 150–200 μm and it is largely the result of filling the fiber granules on the compounding line in the process of extrusion of composites to obtain their granulates.

**Figure 10:**
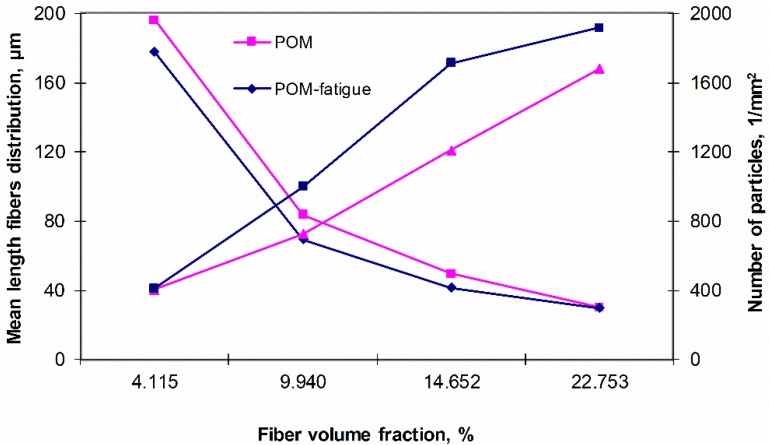
Change in the number of particles and the average fiber length distribution depending on the volume share of (calculated by stereological methods) for composites.

Figure 10 shows the change in frequent quantities and the average fiber length distribution depending on the volume fraction of fibers in the matrix. The results of the basic calculations of stereological parameters (made using computer image analysis methods), present interesting conclusions. During the analysis of polyoxymethylene composites, along with the increase in the calculated volume fraction of fibers, the average fiber length decreases due to the mechanisms that occur during the processing process, which can be directly translate into the average number of particles, additional analysis of composites that are subjected to fatigue loads suggest a further increase in the number of particles in relation to the material not subjected to fatigue loads.

In order to evaluate the effect of the fiber geometry on the properties of poly(oxymethylene) composites, the mechanical properties of POM were compared with a content of 25 wt %. fiber with two different diameters: 10 and 13 μm. The properties determined in the tensile test (Table 4) change slightly. However, for the larger fiber diameter, the properties defined in the tensile and bending tests are higher than properties of composites with diameter of 10 µm. Further research, especially mechanical properties at variable time loads indicate that composites filled with smaller diameter of fibers have better fatigue properties. This phenomenon is associated with several factors, firstly, the beginning of fiber cracking has a place in surface defects, the smaller the diameter of the fiber, the smaller its surface and at the same time the less probability of surface defects conducive to cracking, which contributes to increase strength of the fiber. Secondly, the matrix bond surface should be large enough to increase interphase strength, the smaller the diameter of the fiber and the longer it is, the greater the ratio of the fiber surface to its volume. In addition, in many cases fiber ends carry less load and can act as natural notches, limiting the number of ends increases the ability to carry loads [41,42,43].

**Figure 11:**
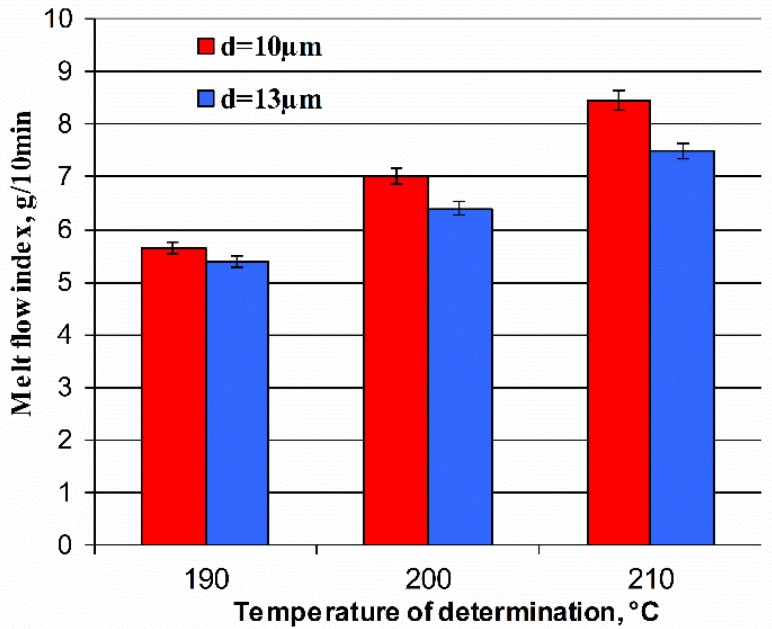
Comparison of the melt flow index of poly(oxymethylene) composites with a 25% addition of glass fiber with diameters about 10 and 13 μm (load P = 2.16 N).

Figure 11 presents the results of the melt flow index for poly(oxymethylene) composites. Regardless of the temperature, fiberglass composites with a smaller diameter of fibers show better processing properties and they are particularly suitable for the injection of products with complex shapes and small sizes. In this case, it is also worth considering a higher injection temperature due to the better melt flow.

**Figure 12:**
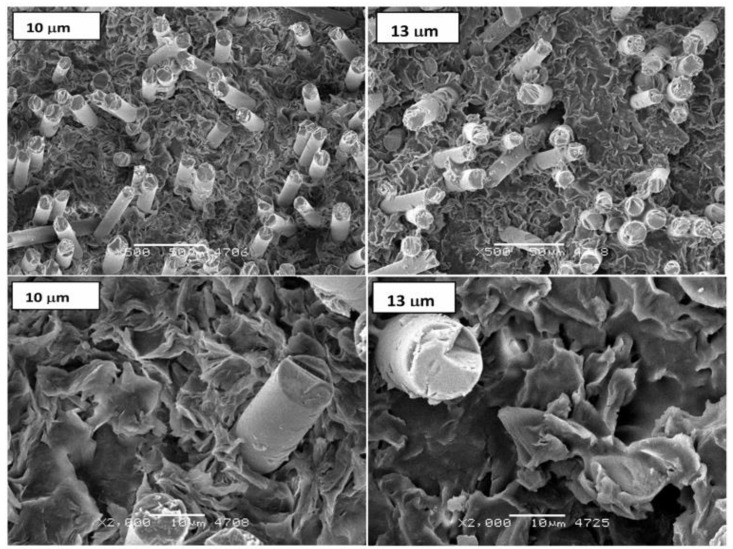
Characteristic microstructure images of selectively etched samples for POM composites with 25 wt %. of fiber with different diameters (ø 10 and 13 µm).

**Figure 13:**
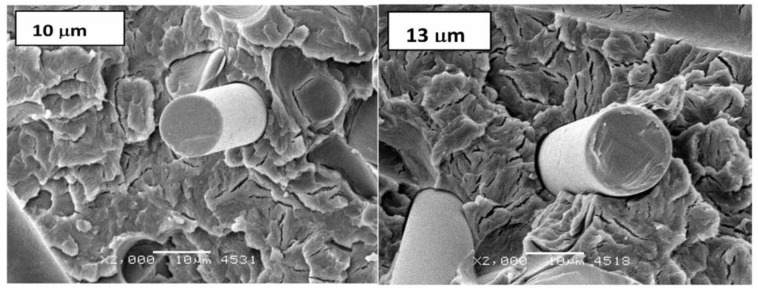
Images of spherulites with lamellar structure and small share of ductile fracture at the breakthroughs obtained at ambient temperature.

In the microstructural image (Figure 12) of selectively etched samples, a reduction in the dimensions of single spherulites for a composite reinforced by fibers with a diameter of 10 μm and a larger leaching of the amorphous phase for a composite with a fiber with a diameter of 13 μm can be noticed. In the photos of breakthroughs obtained at ambient temperature (Figure 13), similar effects of spherulite fragmentation with preserved plate structure in the shape of developed rosettes can be observed. Furthermore, a small share of a ductile fracture (due to the high crystallinity of poly(oxymethylene) and fine crystalline structure are presented.

**Figure 14:**
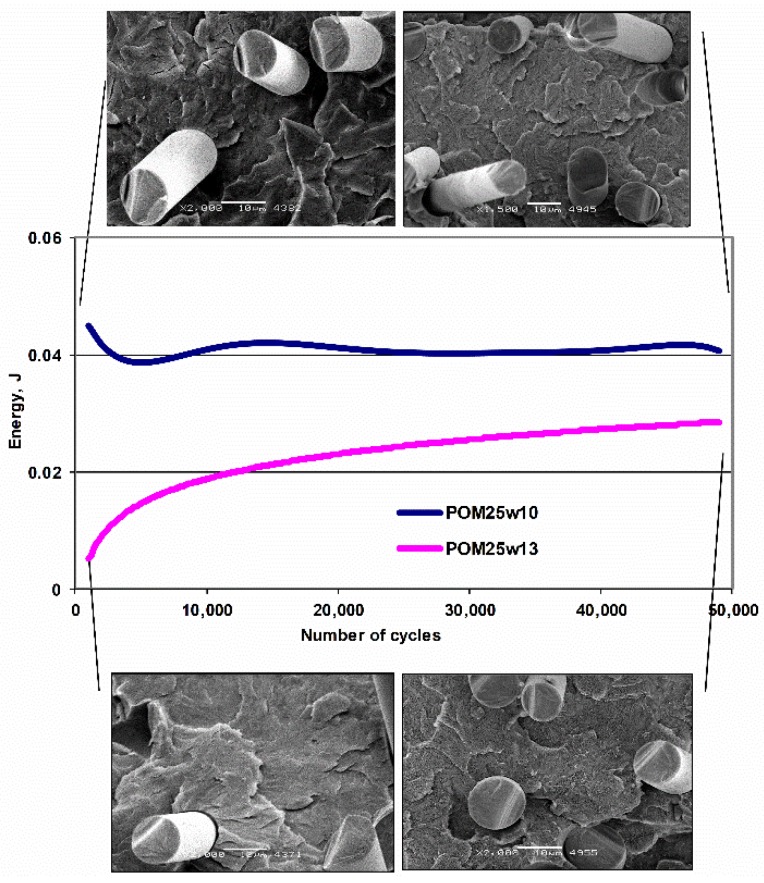
Change of dispersed energy in one cycle of POM composites with 25% fiber content with different diameters (ø 10 and 13 µm).

Figure 14 shows the change in dissipated energy in cycle 1 as a function of the number of cycles for POM composites with different diameters of the fibers. Those containing fibers with a diameter of 10 µm show a greater and constant ability to dissipate energy, increasing their fatigue strength. In images of breakthroughs after fatigue, an increase in the proportion of ductile phase and smaller sizes of spherulites are shown. Which promotes the formation of more nucleation seeds and at the same time due to the larger number of particles, partly blocking its development.

**Figure 15:**
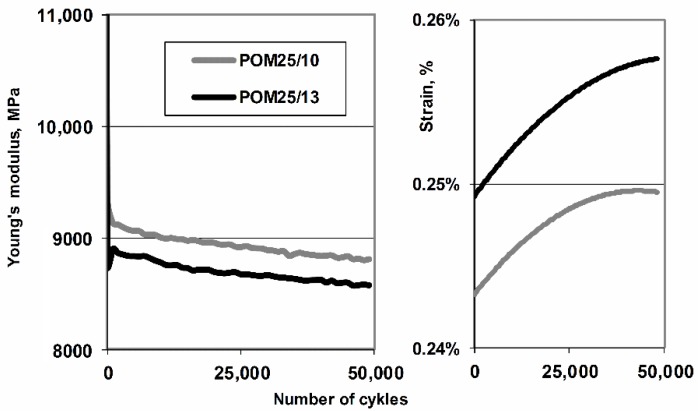
Comparison of the secant modulus of elasticity and mean strain calculated in each loop (50,000 cycles, 5 Hz) for acetal composites filled with 25% glass fiber with two different diameters.

**Figure 16:**
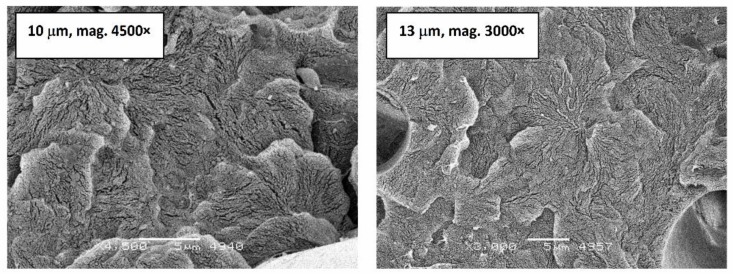
Images of spherulites with lamellar structure in the shape of developed rosettes on the surfaces of breakthroughs in N_2_ in composites after fatigue.

The comparison of modulus of elasticity and medium strain for acetal composites filled with 25% glass fiber with two diameters are presented in Figure 15. Chart confirms the better ability (about 10% improvement) of fibers with smaller diameter to carry the fatigue load by the composite. Lower module drops (despite the larger “static” module for a composite with fiber diameter of 13 μm) and smaller deformation (at the same load) for a fiber-filled composite with a smaller diameter (10 µm) predispose this type of fiber to create composites for applications subjected to dynamic loads, additionally, it should be taken into account that the density of composites does not change with mass rather than volume fraction Figure 16 shows the images of breakthroughs in N_2_ of composites after fatigue. Micropictures present structure with characteristic features of spherulite with a plate-like structure in the shape of developed rosettes and considerable fragmentation resulting from fatigue. The smaller diameter of the fiber results in a structure with stronger fragmentation, but a greater degree of ordering capable of long-term transfer of dynamic loads.

**Figure 17:**
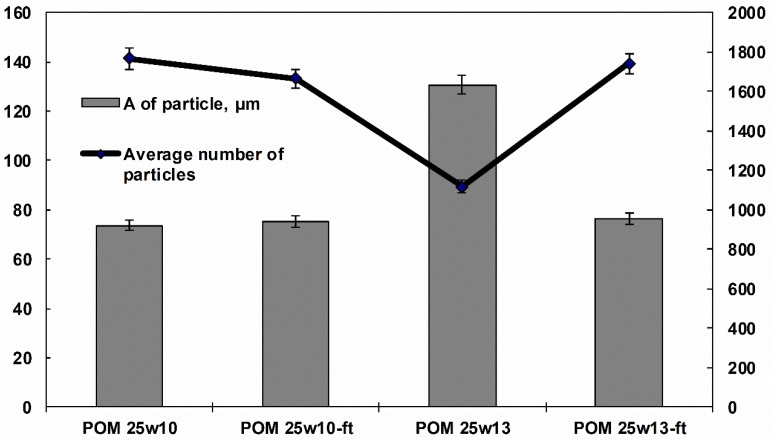
Comparison of the number of glass fiber crossection on the area unit NA particles and average areas of glass fiber crossection calculated for acetal composites filled with 25% glass fiber with two diameters and subjected to fatigue load.

The computerized stereological calculation (presented in Figure 17) results show that in the case of composites with a larger diameter of fiber, it is easier to break them under the influence of fatigue loads, as a consequence of grow average number of particles and drop in their average area. Filler parameters with a larger diameter after fatigue process reach ranges of those with a smaller diameter for which this process does not lead to significant changes, which indicates their “optimality” and greater ability to carry dynamic loads.

Based on the analysis of the poly(oxymethylene) literature [44,45], it can be assumed that it crystallizes in the hexagonal system (phase α). The description of the crystalline phase of poly(oxymethylene) is presented in Table 5.

**Figure 18:**
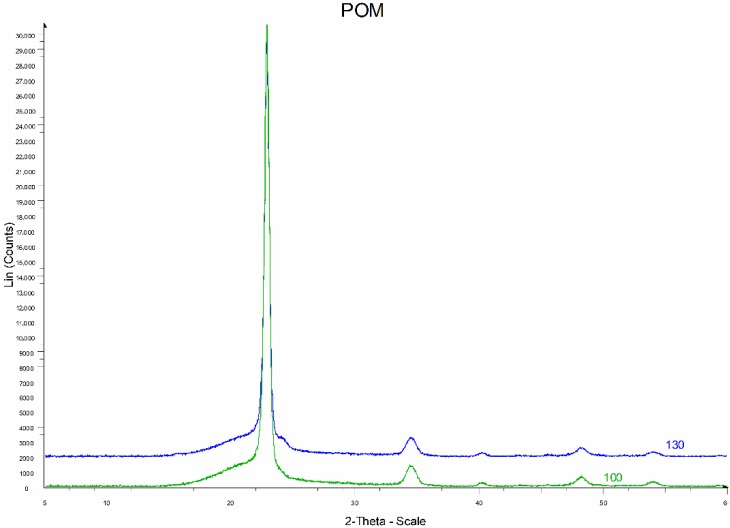
X-ray diffractograms of POM composite with 25% glass fiber with a fiber diameter of 10 and 13 μm. The graphs were spaced along the radiation intensity scale.

The WAXS diffractograms of a poly(oxymethylene) composite with 25% glass fiber with different diameters are shown in Figure 18.

The diffractograms of POM composite samples with 25% glass fiber with different diameters are similar (Figure 18). There are slight shifts of the deflection angle 2Ɵ for some peaks and changes in their intensity. Table 6 lists the peak parameters from the crystalline phase of the POM composite with 25% glass fiber with a diameter of 10 μm (POM25-10) and 13 μm (POM25-13).

The crystalline phase of POM exhibits a strong reflex of 100 at an angle of 2Ɵ = 22°54′ and much weaker reflections 105, 110, 115. Peaks on the diffraction patterns of the tested composites occur at the same angles of deflection as the peaks appearing on the diffraction patterns presented in the literature [44]. During the crystallization of the tested POM composites, a crystalline phase is formed with a network of hexagonal elementary cells.

Thermal analysis of the POM composites was performed and its results are summarized in Table 7. It was assumed that the heat of fusion of the completely crystalline poly(oxymethylene) is Δhc = 247.0 J/g [35].

It is known from the literature that the melting point of poly(oxymethylene) without fillers is *T*_m_ = 183 °C, the glass transition temperature *T*_g_ = −80 °C and the degree of crystallinity *w*_c_ = 80%–90% [37].

Thermal analysis by differential scanning calorimetry was performed using a DSC Q2000 TA apparatus (TA Instruments, New Castle, DE, USA). The samples were heated, cooled, and re-heated at a rate of 10 °C/min, in the temperature range from −80 °C to +300 °C. The melting point (*T*_m_) was determined as the value corresponding to the peak extreme of a given transformation.

**Figure 19:**
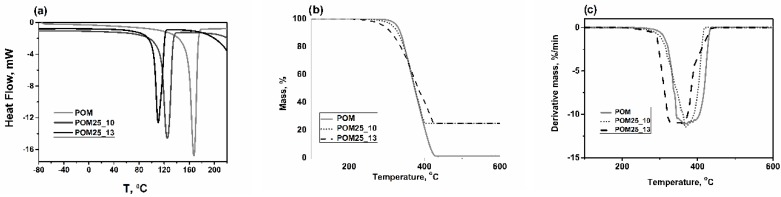
Compilation of calorimetric curves: (**a**) The melting temperature, (**b**) DTG, and (**c**) TGA curves investigated under nitrogen atmosphere. DTG: differential thermal gravimetric; TGA: thermal gravimetric analysis.

Figure 19a shows the calorimetric curves of poly(oxymethylene) and composites containing glass fiber in an amount of 25 wt %. with a diameter of 10 and 13 µm. On the curves, during heating, endothermic effects were recorded. The obtained curves show a similar character. Endothermic peaks corresponding to the material melting process in the range of 90–180 °C were observed. The melting point for a composition with a 25% fiber content with a 10 µm diameter was 124 °C, for a sample with a 13 µm diameter of the fiber −110 °C, while for a sample without the filler the melting point was 167 °C.

The thermal stability of polymer composite materials was tested by TG (TGA) analysis. Figure 19b,c show TG and differential TG (DTG) curves of pure polymer matrix and composite materials.

Measurements were carried out for samples without prior annealing at higher temperatures, after conditioning samples at 25 °C for 3 h.

The DTG peak corresponds to the initiation of material decomposition and thermal degradation of the material, which is useful in engineering applications in determining the operating temperature range. The obtained results indicate that the addition of a filler affects the thermal stability of the polymer matrix; The increased content of residual char (b) in composites strictly corresponds to the filler content. The highest thermal stability can be observed for the POM25_10 sample, which corresponds to a glass fiber composite with a diameter of 10 µm.

## 4. Conclusions

The methodology used in this paper allowed to observation of phenomena and changes in mechanical properties occurring during material fatigue. There is no similar methodology in the world literature combining current analysis of mechanical properties with computer analysis of breakthrough images at various stages of material fatigue. A comparison of fiberglass composites with carbon fiber reinforced composites suggests less adherence of glass fibers to the poly (oxymethylene) matrix. Nevertheless, the use of glass fibers almost doubles the modulus of elasticity between 10% and 20% by weight of the fiber content relative to the unreinforced material. Further increase in fiber content does not have such a significant impact on the value of Young’s modulus. However, as the fill increased, a synergistic increase in tensile strength was observed. These results suggest that depending on the application, it is possible to modify POM-based composites that will have optimal strength parameters. In addition, the results of impact tests carried out especially in a wide temperature range indicate an increase in impact resistance already at 10% of the fibers by weight, and testing of mechanical hysteresis loops reveals an increase in the ability to dissipate energy and transfer an increasing number of loads, which ensures an extended life time in real, variable working conditions.

Fiber geometry analysis showed that composites with a smaller fiberglass diameter have better fatigue and processing properties. Assessment of the impact of the size and type of reinforcement is very important because it finds reference in almost every examined material property, e.g., mechanical, thermal, tribological, optical, and chemical properties, which are well known in many works in the field of polymer composites as well as metal and ceramic composites [46,47].

The presented research results suggest that poly(oxymethylene) glass fiber composites, especially those with a small diameter (about 10 µm), can be successfully used for components with complex shapes that are subject to changes in dynamic loads changing over time, ensuring very good strength parameters. Designing, producing, and testing new materials with increased mechanical performance is extremely important because it affects not only the construction sector as the introduction of new advanced materials to the market, but the extension of the explant time limits the material production, which indirectly also affects the environment, which is immeasurable charged in production processes emitting harmful gases into the atmosphere [48,49,50].

## Figures and Tables

**Table 1:** Comparison of basic processing physical properties of poly(oxymethylene) (POM) composites filled with glass and carbon fiber.

Fibers Content, %	Melt Flow Index, g/10 min		Density, g/cm^3^	
	Fibers Glass	Carbon Fibers	Fibers Glass	Carbon Fibers
**0**	13.0	27.0	1.41	1.41
**5**	-	12.5	-	1.42
**10**	10.0	10.5	1.48	1.43
**20**	8.0	6.5	1.55	1.44
**30**	6.0	4.5	1.63	1.46
**40**	4.5	-	1.70	-

**Table 2:** List of selected test results of POM composites with glass and carbon fibers obtained during bending tests and Charpy impact tests.

Fibres Content, %	Flexural Modulus, MPa		Energy at F_max_, J		Total Strain, %		Charpy Impact Strength, kJ/m^2^	
	Fibers Glass	Carbon Fibers	Fibers Glass	Carbon Fibers	Fibers Glass	Carbon Fibers	Fibers Glass	Carbon Fibers
**0**	2120	2120	2.95	2.95	50	30	30	30
**5**	-	5150	-	3.55	-	3.10	-	23
**10**	3460	8090	2.25	3.05	2.60	2.20	35	22
**20**	5380	14,020	1.55	1.90	1.55	1.20	37	22
**30**	7420	16,950	1.45	1.90	1.35	1.15	38	18
**40**	10,140	-	1.00	-	1.00	-	38	-

**Table 3:** Accepted load ranges (0.1–0.6 P_max_) and modules of tested POM composites with glass fiber and calculated average value of dispersed energy E_dys_ in each cycle.

Fibres Content, %	Load Range, kN	E, MPa	E_dys_, J
**0**	0.13–0.97	3025	0.011
**10**	0.23–1.60	5000	0.022
**20**	0.26–1.80	9000	0.021
**30**	0.29–1.90	11,900	0.026
**40**	0.33–2.40	14,350	0.026

**Table 4:** Comparison of basic mechanical properties of poly(oxymethylene) composites with a 25% addition of glass fiber with 10 and 13 μm fiber diameters.

Diameter of Fiber	ø = 10 μm	ø = 13 μm
**Young’s Modulus E, MPa**	10,500	12,200
**Tensile Strength, MPa**	80.0	85.0
**Strain at Break, %**	1.0	1.0
**Energy at P_max_, J**	0.80	0.85
**Unnotched Impact Strength, kJ/m^2^**	9.6	11.3

**Table 5:** The description of the crystalline phase of poly(oxymethylene).

Crystalline Phase	Diffraction Angle 2Ɵ,°	Diffraction Reflex	The Distance between Planes d_hkl_, Å
**α**	22°54′	100	3883
	34°36′	105	2592
	40°16′	110	2239
	48°28′	115	1874

**Table 6:** Description of the peaks with the highest intensity on the diffraction patterns of POM composite with 25% glass fiber with a diameter of 10 and 13 μm.

POM 25-10		POM 25-13	
Diffraction Angle 2Ɵ, °	Diffraction Reflex	Diffraction Angle 2Ɵ, °	Diffraction Reflex
22°54′	100	22°54′	100
34°36′	105	34°36′	105
40°16′	110	40°16′	110
48°28′	115	48°28′	115

**Table 7:** Results of thermal analysis of POM composite with 25% glass fiber with a diameter of 10 and 13 μm.

Index	*T*_m_, °C	*w*_c,h_, %
**POM**	167	82
**POM 25-10 µm**	124	65.0
**POM 25-13 µm**	110	47.0
